# Autosomal and uniparental portraits of the native populations of Sakha (Yakutia): implications for the peopling of Northeast Eurasia

**DOI:** 10.1186/1471-2148-13-127

**Published:** 2013-06-19

**Authors:** Sardana A Fedorova, Maere Reidla, Ene Metspalu, Mait Metspalu, Siiri Rootsi, Kristiina Tambets, Natalya Trofimova, Sergey I Zhadanov, Baharak Hooshiar Kashani, Anna Olivieri, Mikhail I Voevoda, Ludmila P Osipova, Fedor A Platonov, Mikhail I Tomsky, Elza K Khusnutdinova, Antonio Torroni, Richard Villems

**Affiliations:** 1 Department of Molecular Genetics, Yakut Research Center of Complex Medical Problems, Russian Academy of Medical Sciences and North-Eastern Federal University, Yakutsk, Russia; 2 Department of Evolutionary Biology, University of Tartu, Tartu, Estonia; 3 Estonian Biocentre, Tartu, Estonia; 4 Institute of Biochemistry and Genetics, Ufa Scientific Center, Russian Academy of Sciences, Ufa, Russia; 5 Department of Anthropology, University of Pennsylvania, Philadelphia, USA; 6 Dipartimento di Biologia e Biotecnologie, Università di Pavia, Pavia, Italy; 7 Institute of Internal Medicine, Siberian Branch of Russian Academy of Medical Sciences, Novosibirsk, Russia; 8 Institute of Genetics and Cytology, Siberian Branch of Russian Academy of Sciences, Novosibirsk, Russia; 9 Institute of Health, North-East Federal University, Yakutsk, Russia; 10 Department of Genetics and Fundamental Medicine, Bashkir State University, Ufa, Russia; 11 Estonian Academy of Sciences, Tallinn, Estonia

**Keywords:** mtDNA, Y chromosome, Autosomal SNPs, Sakha

## Abstract

**Background:**

Sakha – an area connecting South and Northeast Siberia – is significant for understanding the history of peopling of Northeast Eurasia and the Americas. Previous studies have shown a genetic contiguity between Siberia and East Asia and the key role of South Siberia in the colonization of Siberia.

**Results:**

We report the results of a high-resolution phylogenetic analysis of 701 mtDNAs and 318 Y chromosomes from five native populations of Sakha (Yakuts, Evenks, Evens, Yukaghirs and Dolgans) and of the analysis of more than 500,000 autosomal SNPs of 758 individuals from 55 populations, including 40 previously unpublished samples from Siberia. Phylogenetically terminal clades of East Asian mtDNA haplogroups C and D and Y-chromosome haplogroups N1c, N1b and C3, constituting the core of the gene pool of the native populations from Sakha, connect Sakha and South Siberia. Analysis of autosomal SNP data confirms the genetic continuity between Sakha and South Siberia. Maternal lineages D5a2a2, C4a1c, C4a2, C5b1b and the Yakut-specific STR sub-clade of Y-chromosome haplogroup N1c can be linked to a migration of Yakut ancestors, while the paternal lineage C3c was most likely carried to Sakha by the expansion of the Tungusic people. MtDNA haplogroups Z1a1b and Z1a3, present in Yukaghirs, Evens and Dolgans, show traces of different and probably more ancient migration(s). Analysis of both haploid loci and autosomal SNP data revealed only minor genetic components shared between Sakha and the extreme Northeast Siberia. Although the major part of West Eurasian maternal and paternal lineages in Sakha could originate from recent admixture with East Europeans, mtDNA haplogroups H8, H20a and HV1a1a, as well as Y-chromosome haplogroup J, more probably reflect an ancient gene flow from West Eurasia through Central Asia and South Siberia.

**Conclusions:**

Our high-resolution phylogenetic dissection of mtDNA and Y-chromosome haplogroups as well as analysis of autosomal SNP data suggests that Sakha was colonized by repeated expansions from South Siberia with minor gene flow from the Lower Amur/Southern Okhotsk region and/or Kamchatka. The minor West Eurasian component in Sakha attests to both recent and ongoing admixture with East Europeans and an ancient gene flow from West Eurasia.

## Background

With an area of more than three million square kilometers – roughly one third of that of Europe - the Sakha Republic (Yakutia) dominates Eastern Siberia. The southern part of Sakha extends to southern Siberia, which has served as an entry region into northern Asia [1,2]. Southern Siberia connects Sakha with the Inner Eurasian steppe belt, which stretches from the Black Sea to the Yellow Sea and has enabled human movements across large distances from east to west and *vice versa*. The northeastern part of Sakha overlaps with former Beringia, which connected Asia and America during the Last Glacial Maximum (LGM), permitting human migration to the Americas [3,4]. Because Sakha, particularly the Lena valley, served as the main pathway to the arctic coast and, beyond that, America, during Paleolithic times [5], an understanding of its settlement history is important to elucidate the colonization of Northeast Eurasia as well as the peopling of the Americas.

Anatomically modern humans colonized Sakha, including the high Arctic, about 30,000 years ago [6]. Southern Siberia as well as the southern part of Sakha, was continuously populated through the LGM [2,7]. The population began to increase rapidly ~19,000 years ago [8]. Consecutive archaeological cultures in the territory of Sakha hint at multiple waves of migrations from southern areas surrounding the upper reaches of the Yenisey, Lake Baikal and the Amur River [9,10]. Ancient tribes, inhabiting this area since the Neolithic, are regarded as the presumable ancestors of different contemporary circumpolar ethnic groups speaking Paleoasiatic and Uralic languages, while Tungusic-speaking tribes spread all over Siberia at a later time [5,10]. The ancestors of Turkic-speaking Yakuts, under Mongol pressure from the south, moved from the Baikal region up the Lena valley, arriving at the middle reaches of the Lena and Vilyuy Rivers presumably during the 11^th^-13^th^ centuries [5,10,11]. In the 17^th^ century Yakutia was incorporated into the Russian Empire.

The first genetic studies of the native populations of Sakha based on haploid loci (mitochondrial DNA (mtDNA) and non-recombining part of Y chromosome (NRY)) primarily focused on the peopling of the Americas [12,13] and as a “by-product”, detected a very strong bottleneck in Yakut male lineages [14]. Native Siberians, including populations from Sakha, continually receive attention in relation to the colonization of the Americas [15-18], whereas phylogenetic analyses of their uniparental data have added valuable information about the colonization and re-colonization of northeastern Eurasia. The analysis of Siberian mtDNA pool has provided evidence to rule out the existence of a northern Asian route for the initial human colonization of Asia [19], and revealed that the present-day northern Asian maternal gene pool consists of predominantly post-LGM components of eastern Asian ancestry [20,21]. The most frequent Y-chromosome haplogroup in northern Eurasia – N1c – most probably arose in present day China and spread to Siberia after the founder event associated with the human entry into the Americas [22]. Two other Y-chromosome haplogroups dominant in Siberia – C3 and Q1 – are more ancient in northern Asia [17,23].

Genetic studies focused on the Yakuts have shown their strong genetic similarity to South Siberian/Central Asian populations [19,24-28]. MtDNA and Y-chromosome variation in Tungusic sub-groups from different part of northern Asia (Sakha, Middle Siberia and the Russian Far East) has revealed the common shared origin of Evenks and Evens [29]. In addition, mtDNA data of Arctic Siberian populations have shown a genetic discontinuity between Yukaghirs, the oldest population in Sakha, and the adjoining Chukchi, descendants of the latest inhabitants of Beringia [30]. Most of the existing mtDNA data from Sakha populations were obtained by the examination of hypervariable segment I (HVSI) sequences and a limited number of coding region markers, thus permitting to determine the main haplogroups only. However, analyses of large data sets of eastern [31,32] and northern Asian complete mtDNA sequences [19-21,30,33,34] have significantly refined the topology of the mtDNA phylogeny, providing new informative markers for large scale population studies. This was an essential prerequisite to clarify the events that led to the re-colonization of Siberia, as most of the newly defined sub-haplogroups common in Siberia have been dated as post-LGM [20,21,35].

A global study of genetic variance encompassing 51 populations, carried out at the level of 650,000 genome-wide single nucleotide polymorphisms (SNPs) [36], revealed that the Yakuts were closest to the Han Chinese, Japanese and other, less numerous East Asian populations. Even so, Yakuts stand out among East Asian populations due to two distinct signals: the first signal, a minor one, brings Yakuts together with Amerinds, probably reflecting the deep shared ancestry of Siberians and Native Americans, and the second signal is explained by an overlap with the major genetic component in European populations. Analysis of a dataset complemented by eleven more Siberian populations differentiated Siberians from East Asian populations [37]. Furthermore, this analysis separated Koryaks and Chukchi from the rest of the Siberians, demonstrating a close genetic proximity of Yakuts and Evenks. However, the population coverage of Siberia is still limited in genetic studies. For instance, the second Tungusic-speaking population of Sakha – Evens – was not represented in previous analyses. Moreover, Siberian populations have so far not been in the focus of autosomal SNP variance pattern analyses.

In the present study, we combined detailed phylogenetic analyses of the maternal and paternal lineages of the native populations of Sakha with the analysis of a genome-wide sample of more than 600,000 SNPs to better define the genetic relationships between Sakha, South Siberia, East Asia, Northeast Siberia, and Europe, with an emphasis on clarifying the genetic history of the native populations of Sakha. We applied phylogenetic analysis to 829 mtDNAs and 375 Y chromosomes from five populations of Sakha (Yakuts, Evenks, Evens, Dolgans and Yukaghirs) and Dolgans of the adjacent Taymyr Peninsula, and implemented F_ST_, principal component analysis (PCA) and ADMIXTURE to autosomal SNPs in a sample set combining 40 newly genotyped Siberian individuals, published data on Siberia [36,37] and relevant global reference populations [36-38].

## Results

### MtDNA variation among the native populations of Sakha

**Figure 1:**
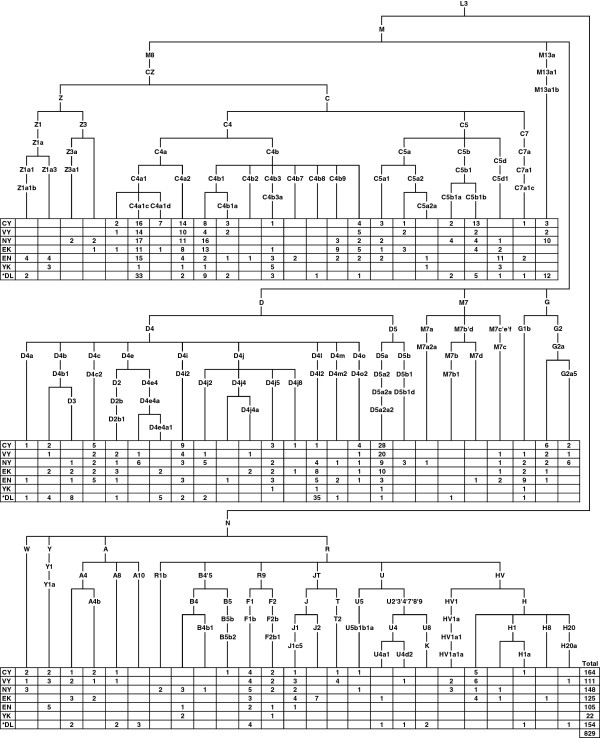
**The phylogenetic relationships of mtDNA haplogroups and their distribution among the native populations of Siberia.** Populations are coded as follows: CY = Central Yakuts, VY = Vilyuy Yakuts, NY = Northern Yakuts, EK = Evenks, EN = Evens, YK = Yukaghirs, DL = Dolgans. *Dolgan samples from Sakha and Taymyr were pooled together.

829 mtDNAs of maternally unrelated individuals from five populations – the Turkic-speaking Yakuts and Dolgans, the Tungusic-speaking Evenks and Evens, and the Yukaghirs who form a small language isolate – were analyzed. High-resolution phylogenetic analysis revealed a total of 147 haplotypes from 73 sub-haplogroups (Figure 1, see Additional file 1 for detailed information). 92% of mtDNAs fall into eastern Asian haplogroups, while western Eurasian lineages constitute a minority. The prevalent haplogroup in Sakha is C, which comprises 43% of the variation in our sample. Haplogroup C is represented mainly by lineages of two major sub-haplogroups – C4 and C5. The only exceptions are the mtDNAs of one Central Yakut, one Dolgan and two Evens which fall into clade C7a1c, the only Siberian branch of the otherwise Northeast Indian specific haplogroup C7. The majority of C clades present in our sample (C4a1c, C4a1d, C4a2, C4b1, C4b3, C5a1, C5b1a, C5b1b and C5d1) are also common among native populations from South Siberia and the Lake Baikal region [20], confirming genetic continuity between Sakha and the southern regions of Siberia. In our sample there are two Evens and one Yukaghir with Koryak-specific maternal lineages – C4b2 and a sub-clade of C5a2 defined by back mutation 16189C and designated as C5a2a in [20]. These C lineages together with G1b, all characteristic of Northeast Siberian populations – Koryaks, Itelmens [39] and Chukchi [30] – , make up only 2% of our sample. Although all native populations from Sakha have uniformly high frequency of haplogroup C, they differ in the proportions of the sub-haplogroups. Yakuts are characterized by a prevalence of C4a1c, C4a2 and C5b1b, whereas Evenks, otherwise very similar to Yakuts, have a higher frequency of the C4 sub-clade defined by the transition T3306C and designated here as C4b9. The sub-haplogroups C4b3a, C4b7 and C5d1 are common for both Evens and Yukaghirs.

The second dominant haplogroup in Sakha is D, encompassing 30% of the mtDNAs in our sample. Haplogroup D4 is represented by a diverse set of low frequency clades, whereas D5 consists almost exclusively of D5a2a2, which reaches its highest frequency among Central and Vilyuy Yakuts (Figure 1). Analyses of ancient DNA from Yakut burials (15^th^-19^th^ century) have revealed similar frequencies of D5a2a2 [40-43]. This raises the possibility that the lineage was brought to the Lena, Amga and Vilyuy valleys by the ancestors of the Yakut people. The presence of D5a2a2 among other populations of Sakha is probably due to gene flow from the Yakuts.

The minor haplogroup Z is represented in our sample by two sub-haplogroups – Z1a was found among Evens, Yukaghirs and Dolgans, whereas Z3 is present among Northern Yakuts and Evenks (Figure 1). Four Z lineages were characterized based on mtDNA complete sequences and represented in a wider phylogenetic context (see Additional file 2). One Even mtDNA shares the transition A11252G with Yukaghirs, forming the clade designated here as Z1a3. The second Even lineage clusters together with Yukaghir and Nganasan into the clade determined by two transitions, G7521A and G8251A, and denoted here as Z1a1b. The time-depth of Z1a (~9,400 years ago) together with Arctic-specific clades Z1a1b, Z1a2a and Z1a3 nested in Z1a (see Additional file 2 and Additional file 3) hints at the possibility that Z1a might have been present in the northern regions of Siberia at least since the Neolithic times. One Yakut lineage falls into the sub-haplogroup Z3a, described so far only in Tibeto-Burman speaking tribal populations of northeastern India [44]. These populations originally descended from ancient tribes of northwestern China and subsequently moved to the south, admixing with southern peoples. Another Yakut Z3 lineage shares the transition G5460A with one Chinese mtDNA (see Additional file 2). Two Northern Yakuts and one Evenk carry this lineage, but none of the rest of Siberian mtDNAs (see Additional file 3). Taken together, these facts suggest that the Z3 lineages have been carried to Sakha relatively recently, most probably by Yakut predecessors.

Besides East Asian maternal lineages, the mtDNA pool of the native populations of Sakha contains a small (8%), but diverse set of western Eurasian mtDNA haplogroups, mostly present among Yakuts and Evenks (Figure 1). The most common western Eurasian haplogroups in Sakha are H and J.

A rare mtDNA haplogroup, R3, was identified in Northern Yakuts by full sequencing. As R3 shares one coding and two control region substitutions with haplogroup R1, it would be more parsimonious to represent R1 and R3 as deep-rooted branches of haplogroup R1 (see Additional file 4). Although np-s 16311 and 16519 are hypervariable and therefore the status of these substitutions in defining R1 is controversial, the transition at np 1391 is much more stable and the motif (1391C-16311C-16519T) consisting of these three substitutions should possess sufficient phylogenetic status. We have designated the former R1 as R1a and the former R3 as R1b. In addition to the Yakut haplotype, R1b encompasses lineages from West Bengal, Armenia and Finland.

### Y-chromosome profiles of the native populations of Sakha

**Figure 2:**
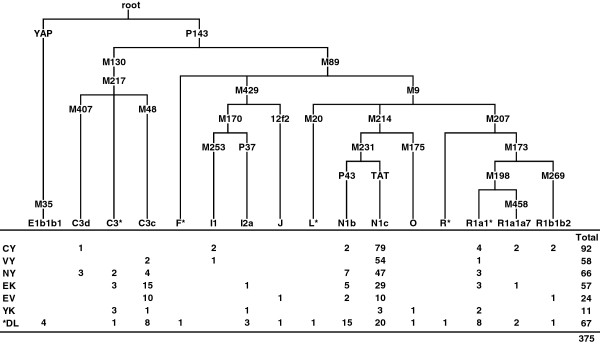
**The phylogenetic relationships of Y-chromosome haplogroups and their distribution among the native populations of Siberia.** The defining SNP markers are shown on the branches. Populations are coded as in Figure 1. *Dolgan samples from Sakha and Taymyr were pooled together.

**Figure 3:**
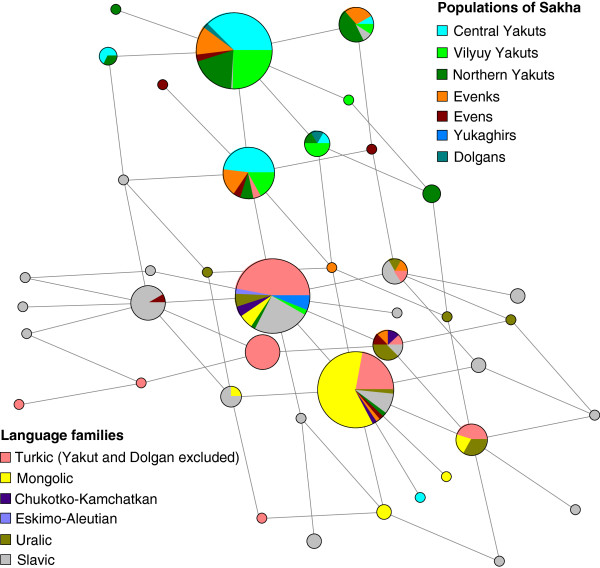
**Phylogenetic network of the Y-chromosome haplogroup N1c.** Median joining network of N1c haplotypes was constructed based on data of five STRs (DYS19, DYS390, DYS391, DYS392 and DYS393) in 398 individuals by using the program Network 4.6.1.0. Circles represent microsatellite haplotypes, the areas of the circles and sectors are proportional to haplotype frequencies according to the data presented in Additional file 6. Populations from Sakha and the linguistic affiliations of the rest of the samples are indicated by color.

The characteristic features of the genetic portraits of native populations from Sakha are much more clearly expressed by paternal lineages (Figure 2, see Additional file 5) than by mtDNA. Yakuts exhibit remarkably low Y-chromosomal genetic diversity because of a striking prevalence of the pan-North-Eurasian haplogroup N1c in their gene pool. Almost all N1c lineages in our sample cluster into a Yakut-specific STR-defined branch (Figure 3, see Additional file 6) established by earlier studies [22,45,46]. The fact that this branch also comprises lineages from the Evenks, Evens and Dolgans may hint at a limited male gene flow from Yakuts to neighboring populations. The Yakut-specific branch stems from a haplotype most common in Tuvinians and Tofalars from the east Sayan region that is found also in some other Siberian and eastern European populations. The sub-cluster of N1c (N3a1 in [46]), which encompasses the Yakut-specific branch, had its first expansion in South Siberia at the boundary of Pleistocene and Holocene [46]. The Yakut-specific clade started to diversify only ~1.6 kilo years ago (kya). Four N1c chromosomes in our sample fall into a cluster comprised mainly of Buryats [46]. It might hint at a gene flow from the predecessors of Buryats in the Lake Baikal region. Besides N1c, Yakuts as well as Dolgans, Evenks and Evens harbor N1b (Figure 2), which achieves its highest frequency in Nganasans [47] and is common among Tofalars, Khakassians, Tuvinians and Shors from South Siberia [46].

Haplogroup C3 is present at 30-40% in Evenks, Evens and Yukaghirs, at 13% in Northern Yakuts and Dolgans and has a minor frequency among Vilyuy and Central Yakuts (Figure 2). The Evenk and Even gene pool encompasses C3c STR haplotypes (see Additional file 5) that partly overlap with those found in more southern populations: the Mongolic-speaking Buryats and Mongolians, the Turkic-speaking Tuvinians and Altaian Kazakhs as well as the Tungusic-speaking Manchus [23,48]. The sub-haplogroup C3d present among Yakuts is very frequent among Buryats, Sojots and Khamnigans from the Baikal region [23]. The C3* lineages of Evenks (see Additional file 7) coincide with the haplotype common among Mongolians [49], probably hinting at a relatively recent male gene flow from Mongols to Evenks. In contrast, the Yukaghirs’ C3* lineages are closest to the Koryak gene pool (see Additional file 8 and Additional file 9). This finding points to the possible common ancestry of at least some paternal lineages in these neighboring populations.

The general pattern of the geographic dispersal of haplogroup Q in Siberian populations is very variable, ranging from high frequency in some populations (Kets and Selkups) to low frequency or total absence in others [17,47]. It could point to the effect of pronounced genetic drift that affects small and diffusely located populations, especially in case of the Y chromosome due to its effective population size being at least four times smaller than that of autosomes. In this context it is not surprising that haplogroup Q was not represented in our Yakutian data set (Figure 2).Typical European and Near Eastern haplogroups R1, I, E1b1b1a and J are present in Sakha at low frequencies. Haplogroup O, common in South-East Asia, was found in only one Yukaghir and one Dolgan (Figure 2).

### Autosomal SNP variance pattern in the native populations of Sakha and beyond

**Figure 4:**
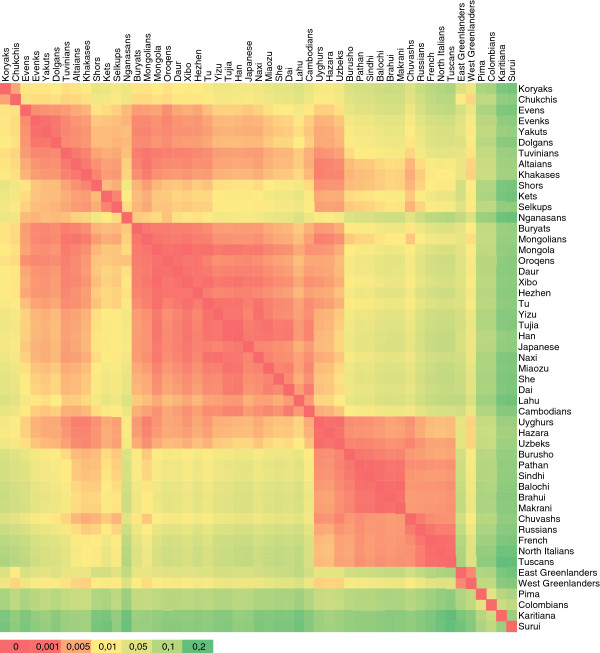
**Heatmap plot of F**_**ST **_**distances.** Only populations with N≥4 are included (see Additional file 10 for list of samples).

Analyses of more than 600,000 common single nucleotide polymorphism loci from the nuclear genome were based on a sample of 758 individuals from 55 populations. The sample set includes 40 previously unpublished samples from Siberia and compatible published data from Europe, Asia and America [36-38] to represent the regional context (see Additional file 10). In the heatmap plot of F_ST_ distances (Figure 4), populations from Sakha form a cluster of low genetic distance, with Yakuts and Evenks being the closest. The smooth transition from Sakha to South Siberia and on to East Asia contrasts with the discontinuity between Sakha and Northeast Siberia.

**Figure 5:**
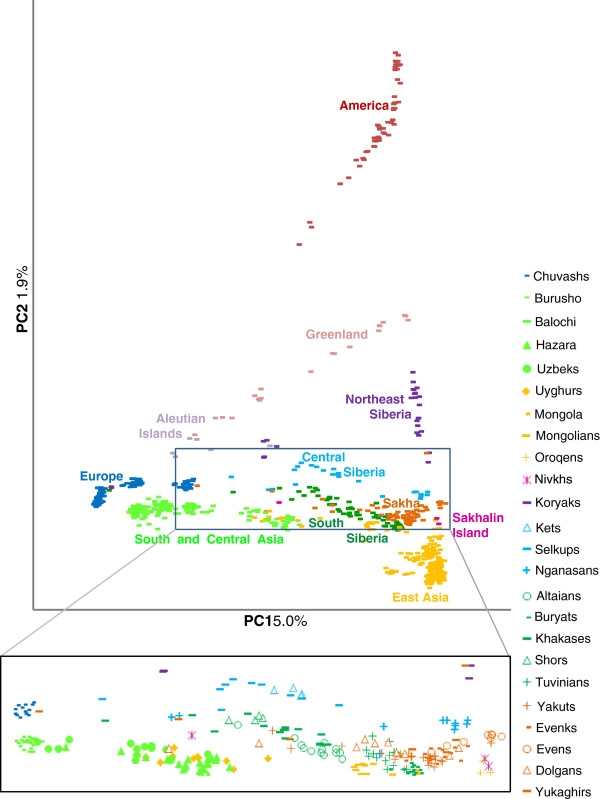
PCA of the native populations of Sakha in the context of other Eurasian and American populations.

The PC analysis demonstrates the clustering of populations from Sakha close to Tuvinians and Buryats from southern Siberia as well as to Mongolians from Mongolia, whereas they are separated from the neighbouring Chukchi and Koryaks (Figure 5). It is remarkable that Nganasans who live in relative isolation on the Taymyr Peninsula are situated near the populations of Sakha in the PC plot. Evenks and Yakuts together with some Dolgans, Evens and Yukaghirs cluster close together, while the rest of the Dolgans, Yukaghirs and a few Yakuts are dispersed between Siberian/Central Asian and one Yukaghir even among East European populations. The majority of Evens form a distinct cluster near the Yakut/Evenk and Nganasan groups.

**Figure 6:**
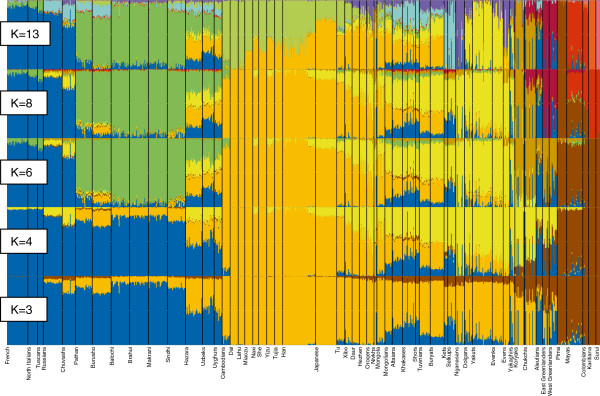
**ADMIXTURE plots.** Ancestry proportions of the 758 individuals studied (from 55 populations) as revealed by the ADMIXTURE software at K = 3, K = 4, K = 6, K = 8, and K = 13.

In order to study the genetic relationships of the populations in more detail, we used the model-based algorithm ADMIXTURE [50] that computes quantitative estimates for inferring the ancestry of individuals from K number of constructed ancestral populations. As expected at K=3 Siberian and East Asian ancestry palettes are largely indistinguishable (Figure 6, see Additional file 11 and Additional file 12). However, there is a minor signal that unites Siberians with Amerinds and Greenlanders, probably reflecting deep-rooted shared ancestry of Siberians and Native Americans. As previously shown [36,37], starting from certain K values (here at K=4), the dominant East Asian – Siberian ancestry component (yellow) splits into two. Lemon yellow is present among northern Asian populations as well as Greenlanders and Aleuts and dark yellow is characteristic of Hans and southern Chinese populations. At K=6, the lemon yellow splits further to discriminate the Greenland and Aleutian populations from the Siberians. Importantly, this new component (olive) is also present among the Chukchis and Koryaks, likely testifying to some level of Beringian continuity. Starting from K=8, Greenlanders acquire their own color, largely restricted to them only. At K=13, a new ancestry component (lavender) appears among the Siberian populations. It is most prominently apparent among the Nganasans and is present as a minor signal in almost all Siberian and northern Chinese populations. Although the level of variation in log-likelihood scores (LLs) within a fraction (10%) of runs with the highest LLs shows that the global maximum is probably not reached at K = 13, seven runs from ten exhibit this new ancestry component (see Additional file 12 and Additional file 13). In the Siberian ancestry palette there is another minor signal, explained by an overlap with the major component in European populations. This component, accounting for about 20% of the ancestry of individuals from South Siberia, decreases practically to zero in northern Siberian populations. However, a few individuals from small northern populations (and, incidentally, all of the Aleuts in our sample) show an exceptionally high proportion of the European component.

The main feature of the autosomal admixture pattern of the native populations of Sakha is the prevalence of the lemon yellow component (Figure 6), especially in Yakuts and, in a smaller extent, in Evenks and Dolgans. The second Siberian-specific component (lavender) makes up approximately two third of the ancestry of Evens, but is barely present in Yakuts. Evens and Yukaghirs show a minor signal overlapping with the dominant component in Koryaks and Chukchis. It most probably reflects a limited gene flow from Northeast Siberia. The presence of the “European” (blue) component in the native populations of Sakha likely testifies to admixture that is recent and/or ongoing, or has been considerable in the past. Although the ancestry panels do not offer specific time estimates for putative admixture events, it is reasonable to suggest that “old” admixture or shared ancestry would even out within a population, whereas more recent and ongoing admixture events would exhibit with large differences between individuals in a population. The latter can most obviously be seen among the Yukaghirs.

### Mantel correlation tests

Mantel tests were used to clarify the role of geography and linguistic diversity in the formation of the genetic variation spatial patterns in northern Asia. We started by analyzing the Altaic-speaking populations, the most numerous and widespread populations in northern Asia. The term “Altaic” has been used here to denote a language family that includes the Tungusic, Turkic and Mongolic languages [51]. A Mantel test performed on 18 Altaic-speaking populations from Siberia, Central Asia, Mongolia and northern China (see Additional file 10) revealed a statistically significant positive correlation between autosomal SNP variation and geography (r = 0.68, P < 0.001) (see Additional file 14). Geography explains 46.8% of the genetic variance. Additional Mantel tests applied to mtDNA and Y chromosome data of 22 Altaic-speaking populations (see Additional file 15) show a correlation between mtDNA variation and geography (r = 0.55, P < 0.001), whereas Y-chromosome variation exhibits a weak partial correlation with linguistic data (r = 0.18, P = 0.014) (see Additional file 14). Isolation by distance is the most plausible explanation for the positive correlation between maternal genetics and geography. Patrilocality and a patrilineal clan structure combined with strict exogamy common among Altaic-speaking populations [52] could cause a greater degree of female than male admixture and/or the adoption of languages by females to a greater extent than by males. The correlation between paternal genetics and linguistics hints at the concordant spread of genes and languages, but this has not been the sole process, because linguistic data explain only 3.3% of the Y-chromosome variance. When the Chukchis and Koryaks, representatives of the Chukotko-Kamchatkan language family from Northeast Siberia, were added to the analysis, a considerable partial correlation between autosomal and linguistic variation appeared (r = 0.55, P = 0.002) in addition to the correlation between genetics and geography. Both linguistics and geography explain approximately one third of the genetic variance. The results of the Mantel test based on mtDNA data are very similar (see Additional file 14). In contrast, there is no correlation between paternal genetics and geographic distances or linguistic diversity.

## Discussion

In the following section, we discuss (1) the role of South Siberia in the formation of the existing genetic variation in Sakha, (2) the genetic discontinuity between Sakha and Northeast Siberia, and (3) the origin of the West Eurasian component in the gene pool of the native populations of Sakha.

### Sakha as an extension of South Siberia

The PCA plot and F_ST_ values based on autosomal SNP data demonstrate the genetic proximity of the populations of Sakha to the geographically and linguistically neighboring populations from South Siberia, particularly Tuvinians and Buryats. The ADMIXTURE analysis reveals that the genetic heritage of the populations of Sakha in its great majority is characterized by Siberian-specific ancestry components which stem from East Asia. The Siberian components, comprising the component denoted by lemon yellow that achieves its highest proportion in Yakuts and the one denoted by lavender represented most clearly in Evens, connect the inhabitants of Sakha with southern Siberian populations. Similarly, phylogenetic analyses of uniparental data show that East Asian mtDNA and Y-chromosome haplogroups form the main part of Sakha gene pool, and the majority of maternal and paternal lineages found in Sakha (Figure 1 and 2) are nested in the larger genetic variation of South Siberia [19,20,23,46]. In addition, a fair number of mtDNA haplogroups (C4a1c, C4a1d, C4a2, C4b1, C4b3, C5b1a, D4i2, D4j2, D4j4, D4j5, D4j8, D4o2) as well as Y-chromosome haplogroups C3c and N1b common in Sakha have been dated to the Siberian Neolithic [20,23,35,46], the beginning of which coincided with the period of climatic optimum in the Postglacial. These results all suggest that a substantial part of maternal and paternal lineages in Sakha might stem from Neolithic expansions in South Siberia, having been carried to Sakha afterwards at different times by different peoples. Y-chromosome haplogroup C3c, the most frequent among Evenks and Evens in Sakha (Figure 2, see Additional file 5), might have arrived with the ancestors of the Tungusic peoples. This scenario is supported by the observation that C3c is much more frequent among various Tungusic-speaking peoples from Siberia and northern China than in their neighbors [47].

MtDNA haplogroups Z1a and C4b could represent traces of more ancient migrations in northern Siberia, as these haplogroups have been dated as older and, furthermore, their known sub-clades are found almost exclusively in Arctic populations (see Additional file 2 and Additional file 3) [20,35]. Z1a stretches all over Siberia, but three distinct sub-clades (Z1a1b, Z1a2a and Z1a3) are represented mainly in the northern regions. Interestingly, all three clades encompass the Yukaghirs – nowadays a very small population residing in northeastern Sakha and Chukotka. Z1a1b includes Nganasans from the Taymyr Peninsula and Evens from Sakha besides the Yukaghirs. Analyses of autosomal data confirm the genetic relatedness of Nganasans, Yukaghirs and Evens (Figures 5 and 6). These results support the scenario that the ancestors of Yukaghirs originate in the Taymyr Peninsula in Neolithic times. In the middle of the second millennium BC, the Yukaghir ancestors spread from the Taymyr Peninsula to the east, under pressure from immigrating groups [10]. In the first half of the second millennium AD, the expansion of Evenks cut the Yukaghirs off from Samoyedic-speaking groups and forced them further east, where they ended up being surrounded by the Chukchis, Koryaks, Evens and the ancestors of Yakuts. Remarkable gene flow between the Yukaghirs and Evens is revealed by mtDNA analysis – in our sample, 71% of maternal lineages are shared between the Yukaghirs and Evens. In addition, the Yukaghirs have acquired a few maternal lineages (Z1a2a, G1b, C5a2a) from the Koryaks.

The clearest and most abundant traces in the Sakha gene pool have been left by the most recent migrations. The prevailing Y-chromosome haplogroup among the Yakuts, N1c, makes up over two thirds of paternal lineages in our sample. The Yakut-specific branch comprises also lineages from Evenks, Evens and Dolgans, most probably due to male gene flow from the Yakuts. The fact that the ancestral STR haplotype of the Yakut-specific clade is present among the Tuvinians, Tofalars and Sojots from the eastern Sayan regions [46] could point to the putative “homeland” of Yakutian N1c predecessors. In addition, at least one maternal lineage, the sub-clade of C4a1c defined by the back-mutation C16298T, may have migrated together with the Y-chromosome haplogroup N1c. This clade, represented in contemporary (see Additional file 1) as well as ancient Yakuts [43], has also been found among the Tuvinians [19]. Close similarities between the Yakut and Turkic languages spoken in the Altai-Sayan region [53], as well as some other aspects of the Yakut culture (e.g., pastoralism, clothing, festivals) [54], point to ancestral ties between the Yakuts and the southern Turkic peoples. These facts suggest that the Yakuts originate from the Altay-Sayan region. On the other hand, some mtDNA (D5a2a2, M13a1b, A8, G2a5) and Y-chromosome haplogroups (C3d, the Buryat-specific cluster of N1c) in the Yakut gene pool are shared with Mongolic populations (Buryats, Khamnigans, Mongolians) from the Baikal area and Mongolia [20,21,23,46]. This fact is consistent with the hypothesis proposed on the basis of archaeological findings, postulating that the Yakuts originate from the ancient Turkic-speaking Kurykan people from the Lake Baikal region [5,10,11]. Estimates of the expansion time of the Yakut-specific clade of Y-chromosome haplogroup N1c support the hypothesis: the clade started to diversify ~1.6 kya, about the same time (6^th^ – 10^th^ century AD) when the culture of the Kurykans flourished on the shores of Lake Baikal. The second expansion of the clade, dated to ~0.9 kya, might coincide with the migration of the Yakuts´ ancestors to the middle reaches of the Lena River in the 11^th^-13^th^ centuries AD. Thus, taking into account the aforementioned facts, the Yakut ancestors most probably originated from the Altay-Sayan region and settled for a time in the Lake Baikal area before migrating northwards along the Lena River.

### Genetic discontinuity between Sakha and Northeast Siberia

Although the genetic heritage of the native populations from Sakha as well as Northeast Siberia lies in its great majority in the common East Asian gene pool, analyses of autosomal as well as uniparental markers revealed the genetic divergence between these neighboring regions. The core of the mtDNA gene pool of the native people of Sakha consists of a number of haplogroup C and D sub-lineages (see Additional file 1). The Koryaks and Chukchis harbor only a few topmost clades of these two haplogroups (C4b2, C5a2a, D4b1a2a and D2a) [30,39], but these lineages are uncommon or even absent in Sakha. The prevailing haplogroups among Chukchis – A2a, A2b and D2a [30] – were not found in Sakha, while G1b, Y1a and Z1a2, common among the Koryaks [39], are present in Sakha at low frequencies. The Koryak-specific maternal lineages found in Sakha most probably suggest a recent limited maternal gene flow from Northeast Siberia. In contrast, the Y-chromosome haplogroups N1c and C3 prevalent in Sakha (Figure 2) are also represented in Northeast Siberia [47,55]. However, the Koryaks [23] and the populations of Sakha (see Additional file 5) do not share Y-STR haplotypes of haplogroup C3, and the N1c haplotypes detected among the Koryaks and Chukchis are quite distinct from those of other Siberians [22,46]. In addition, the presence of the Y-chromosome haplogroup Q1a3a [Q(M3)] among the Chukchis [55] distinguishes them from the populations of Sakha, where this haplogroup has not been found. These results point not so much to recent male-mediated gene flow between these neighboring regions but rather to multiple separate migrations from the same source area. The PCA plot (Figure 5) and F_ST_ values (Figure 4) based on autosomal SNP data also show that the Koryaks and Chukchis are distant from the populations of Sakha. However, ADMIXTURE analysis reveals also a deep-rooted shared ancestry of the inhabitants of Sakha and extreme Northeast Siberia (Figure 6). The genetic data are in good accordance with archaeological findings that demonstrate direct cultural contacts between Kamchatka, Chukotka and Yakutia during the late Paleolithic and Neolithic, and suggest a period of relative isolation for the extreme Northeast only since the second – first millennia B.C. [56].

The strong positive partial correlation between genetic and linguistic variation shown by the Mantel test (see Additional file 14) suggests that the same past population processes have shaped linguistic as well as genetic divergence between Sakha and the Kamchatka-Chukotka region. The present study, as well as previous ones [26,29,43], have revealed the main features of the gene pool of the native populations of Sakha to have been shaped by migrations from South Siberia, in particular by relatively recent expansions of Tungusic- and Turkic-speaking peoples. The Chukchis and Koryaks, inhabitants of the Kamchatka-Chukotka region, are considered to be the descendants of the Neolithic indigenous people of Northeast Siberia [56]. The Chukchi and Koryak languages, together with Kerek and Alutor, form the closely-knit Chukotkan group in the Chukotko-Kamchatkan language family [57], the speakers of which inhabit extreme Northeast Siberia. The Chukotko-Kamchatkan languages have no generally accepted relation to any other language family, but sometimes they are classified together with the Nivkh, Yukaghir and Yeniseian languages among the Paleosiberian languages that are believed to represent a remnant of a much richer linguistic palette of Siberia that existed before the Altaic, Uralic and Indo-European languages expanded across most of Siberia [58]. The prevalent mtDNA haplogroups among the Chukchis – A2a, A2b and D2a [30] – are shared with Greenland Eskimos, Aleuts and a few native populations of North America [15,33,59,60] due to either recent female-mediated gene flow from Eskimos/Aleuts or deep shared ancestry. Similarly, analysis of autosomal SNP data reveals an ancestry component shared between the Chukchis and Greenland Eskimos (Figure 6). MtDNA haplogroups G1b and Y1a, common among the Koryaks [39], and G1b, common among the Chukchis [30], connect them with the Nivkhs from the Sakhalin Island [34]. ADMIXTURE analysis also points to genetic connections between the Chukchis, Koryaks and Nivkhs (Figure 6). In addition, quite a strong relationship between the Nivkh and Chukotko-Kamchatkan languages has been shown [57]. MtDNA haplogroups C4b2 and C5a2a form a part of the “C world” common in South Siberia and Sakha, but their autochthonous nature and coalescence time (~1.2 kya and ~2.6 kya, respectively [20]) hint at a period of isolation from the rest of Siberia. Considering the facts discussed above it is probable that large-scale expansions of Tungusic and Turkic peoples in Siberia, which replaced and/or assimilated ancient aboriginal people in Sakha, as well as the relative isolation between Sakha and Northeast Siberia in the subsequent period, are responsible for the formation of a genetic discontinuity between these neighboring regions.

### Origin of the West Eurasian genetic component in the gene pool of the native populations of Sakha

Although the genetic heritage of the native populations of Sakha is mostly of East Asian-ancestry, analyses of autosomal SNP data as well as haploid loci also show a minor West Eurasian genetic component. The patchy presence of the “European” (blue) component in the ADMIXTURE plot (Figure 6), most pronounced in Yukaghirs, probably testifies to recent admixture with Europeans. In addition, the presence of European-specific paternal lineages R1a-M458, I1 and I2a among Yakuts, Dolgans, Evenks and Yukaghirs likely points to a recent gene flow from East Europeans. Although only individuals with self-reported un-admixed ancestry for at least two generations were included in the study of haploid loci, mistakes in ethnic self-identification cannot be entirely excluded. One of the main sources of gene flow has likely been Russians who accounted for 37.8% of the population of Sakha in 2010 [61]. The migration of Russians (at first mainly men) to eastern Siberia started already in the 17^th^ century, when Yakutia was incorporated into the Russian Empire [62].

The mtDNA haplogroup J detected in the remains from a Yakut burial site dated to the beginning of the 17^th^ century [41], long before the beginning of the settlement of Russian families in the 18^th^ century [63], clearly points to more ancient gene flow from western Eurasia. The presence of haplogroups H8, H20 and HV1a1a among the Yakuts, Dolgans and Evenks (Figure 1) also suggests gene flow other than from Russians, because these haplogroups are rare (H8 and H20) or even absent (HV1a1a) among Russians [64-67], but are common among southern Siberian populations as well as in the Caucasus, the Middle and Near East [19,68-70]. Moreover, the HVSI haplotypes of H8, H20a and HV1a1a in our sample exactly match those in the Buryats from the Buryat Republic [19]. Similarly, the Y-chromosome haplogroup J in Dolgans and Evens very likely testifies to gene flow through South Siberia, as it is present among native South Siberian populations [47,71]. The scenario of ancient gene flow from West Eurasia is supported by ancient DNA data, which show that in the Bronze and Iron Ages, South Siberia, including the Altai region, was an area of overwhelmingly predominant western Eurasian settlement [72,73], and the Indo-European migration even reached northeastern Mongolia [74]. To summarize, the West Eurasian genetic component in Sakha may originate from recent admixture with East Europeans, whereas more ancient gene flow from West Eurasia through Central Asia and South Siberia is also probable.

## Conclusions

The analysis of haploid (mtDNA, NRY) and diploid loci of genome provides further evidence that the genetic heritage of indigenous people of Sakha lies in its great majority in the common East Asian gene pool while West Eurasian influence has been minor. The Turkic-speaking Yakuts retain traces of related populations from the Altai-Sayan region as well as distinctive maternal and paternal lineages, either originating from the Mongolic peoples in the Lake Baikal area or influenced by the long timescale residence in Sakha in close proximity to the Tungusic-speaking Evenks. Genetic data fit well with the linguistic and historical evidence regarding the origin of Yakuts. The Yukaghir gene pool harbor traces of more ancient migration(s) in the arctic regions of Siberia, complemented by recent admixture with Europeans. The Evens, linguistically very close to the Evenks, have also acquired genetic inputs from their geographic neighbors, the Yukaghirs. The European component in Sakha may originate from recent admixture with East Europeans and/or from more ancient gene flow from West Eurasia through Central Asia and South Siberia. The genetic proximity of the native populations of Sakha to South Siberians and the genetic divergence between them and the Chukchis and Koryaks, shown by the present study, suggest that the region of Sakha was colonized by multiple migrations from South Siberia with only minor gene flows from the Lower Amur/Southern Okhotsk region and/or Kamchatka.

## Methods

### Samples for uniparental analysis

**Figure 7:**
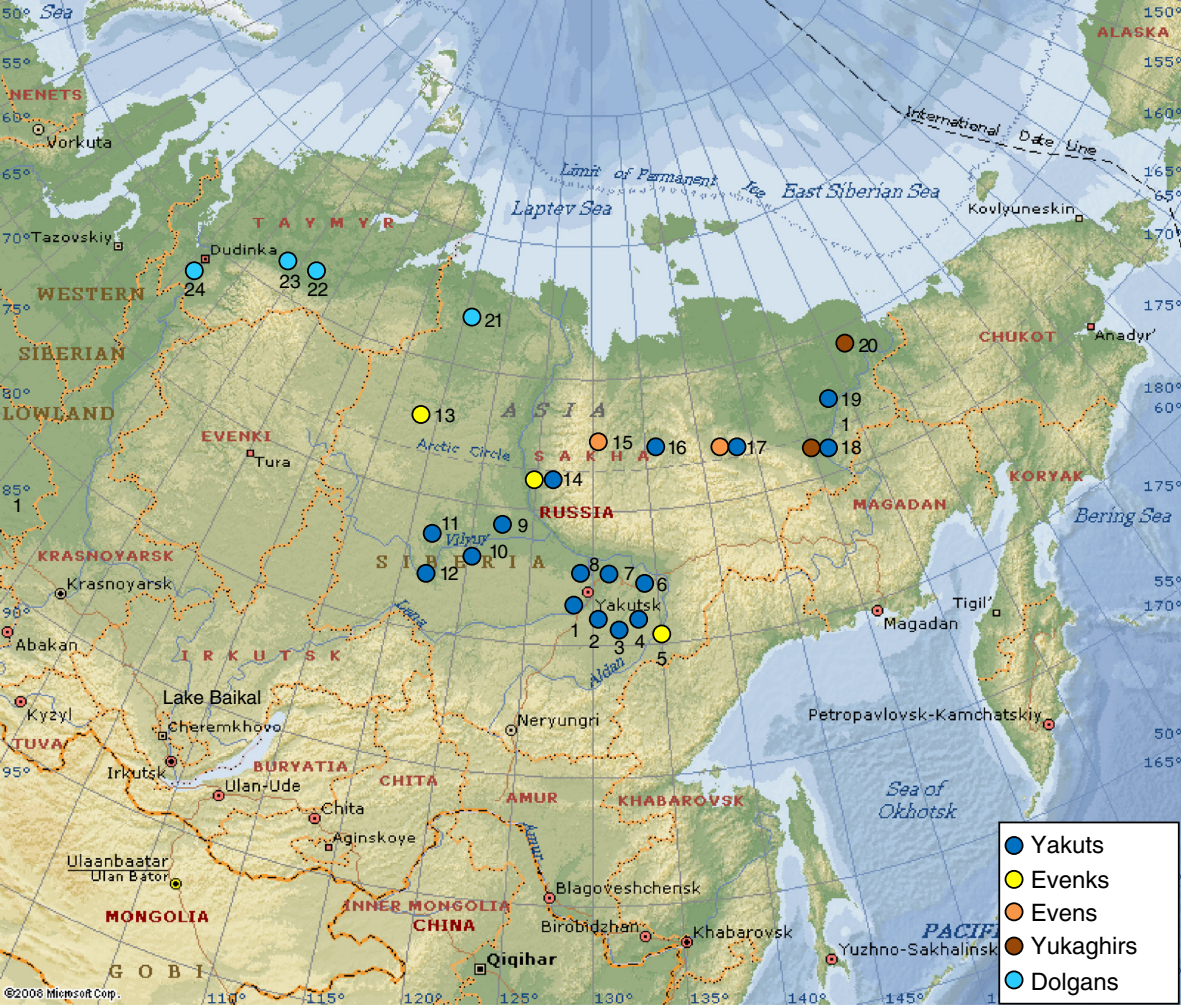
**Map of Sakha, with sampling locations.** Numbers correspond to the sampling locations: 1 – Kangalassky district, 2 – Megino-Kangalassky district, 3 – Amginsky district, 4 – Churapchinsky district, 5 – Ust-Maysky district, 6 – Tattinsky district, 7 – Ust-Aldansky district, 8 – Namsky district, 9 – Vilyuysky district, 10 – Verkhnevilyuysky district, 11 – Nyurbinsky district, 12 – Suntarsky district, 13 – Oleneksky district, 14 – Zhigansky district, 15 – Eveno-Bytantaysky National district, 16 – Verkhoyansky district, 17 – Momsky district, 18 – Verkhnekolymsky district, 19 – Srednekolymsky district, 20 – Nizhnekolymsky district, 21 – Anabarsky district, 22 – Volochanka, 23 – Ust-Avam, 24 – Dudinka.

The sample set comprised 701 Native Siberians from five populations of Sakha: Yakuts, Evenks, Evens, Yukaghirs and Dolgans. The Yakuts have been divided into three ethnogeographic groups: Central, Vilyuy and Northern. The Dolgan sample was complemented by 128 Dolgan DNAs from southern Taymyr. See Additional file 16 for detailed information about the populations studied and the number of samples. Sampling locations are shown in Figure 7. Blood samples were collected from healthy unrelated adult individuals with appropriate informed consent. To eliminate the effect of very recent gene flow on the results of mtDNA and Y-chromosome analyses, only individuals with self-reported un-admixed ancestry for at least two generations were included in the study. DNA was extracted from the blood leukocyte fraction using the phenol-chloroform method [75]. The study was approved by the local ethics committee at the Yakut Research Center of Complex Medical Problems, Siberian Branch of Russian Academy of Medical Sciences. The research has been performed in accordance with the WMA Declaration of Helsinki (59^th^ WMA General Assembly, Seoul October 2008).

### Genotyping

MtDNA haplogroup affiliations of 829 samples were determined by DNA sequencing of HVSI and the screening of 95 coding region markers (see Additional file 1) according to the hierarchy of the mtDNA phylogenetic tree [76,77]. HVSI was sequenced from nucleotide position (np) 16017 to 16399 in all samples. Additionally, 16 coding region markers were examined for 93 haplogroup Z mtDNAs. 21 samples were fully sequenced following a slightly modified published protocol [78]. The preparation of sequencing templates was carried out following standard protocols, employing FIREPol polymerase (Solis BioDyne). Purified products were sequenced with the DYEnamic™ ET terminator cycle sequencing kit (Amersham Pharmacia Biotech) and analyzed on an ABI 3730xl DNA sequencer. Sequences were aligned and analyzed with ChromasPro 1.34 (Technelysium Pty Ltd), and nucleotide mutations were initially ascertained relative to the revised Cambridge Reference Sequence (rCRS) [79]. To record HVSI and complete mtDNA polymorphic positions with respect to the Reconstructed Sapiens Reference Sequence (RSRS) [35], the FASTmtDNA utility provided by mtDNA Community [80] was applied.

Y-chromosome haplogroups were identified for 375 male individuals (including 57 Dolgans from Taymyr) by the analysis of 28 biallelic NRY markers (М9, TAT, SRY1532, 92R7, M207, M20, M89, P43, P37, M231, M35, M78, M269, M412, M458, M48, M52, M70, M130, M170, M173, M175, M201, M217, M253, M407, YAP and 12f2) [81]. Y-chromosome haplogroups were named according to the most recent YCC nomenclature [82,83]. Six microsatellite markers (DYS19, DYS388, DYS390, DYS391, DYS392, DYS393) [84-86] were typed in all Sakha samples and the PCR products were analyzed on the MegaBACE1000 DNA automated sequencer. MegaBACE ET400-R Size Standard was added to each sample for size scaling and the program Genetic Profiler 2.2 (Amersham) was employed for allele scoring. Additionally, 17 STRs were studied in C3* Y chromosomes, using the Y-filer Kit (Applied Biosystems). PCR products were analyzed on ABI 3100Avant genetic analyzer (Applied Biosystems) in the standard fragment analysis protocol mode. GeneScan 500LIZ size standard (Applied Biosystems) was added to each sample for size scaling and GeneMapper 3.5 (Applied Biosystems) employed for allele scoring. Alleles were designated by repeat numbers.

### Phylogenetic analysis

Phylogenetic networks of Y-chromosome haplogroups N1c and C* were reconstructed using the program Network 4.6.1.0, applying the median-joining algorithm [87,88]. The coalescent time of Y-chromosomal N1c haplotypes was calculated according to [89], based on the variability of six microsatellite markers. To estimate the age of mtDNA haplogroups, we calculated rho-statistics (ρ) as average number of substitutions from the root haplotype [90] and its standard deviation (σ) [59]. The mutation rate estimate of 1.665 × 10^-8^ (± 1.479 × 10^-9^) substitutions per nucleotide per year and the calculation approach provided by [91], who transform substitutions to years in a nonlinear manner accounting for the selection effect on non-synonymous mutations, was used to convert the rho-statistics and its error ranges to age estimates in years.

### Samples for genome-wide analysis, genotyping and quality control

Forty samples from nine Siberian populations (Chukchis, Dolgans, Evens, Kets, Khakases, Koryaks, Nivkhs, Shors and Yakuts, see Additional file 10 for detailed information) were genotyped with Illumina 650 K or 660 K SNP array according to manufactures´ specifications. All subjects filled and signed personal informed consents and the study was approved by the scientific council of the Estonian Biocentre. These data were analyzed together with published data from [36-38]. To search for possible close relative pairs among the individuals, we applied software KING (version 1.4) [92] to analyze the entire dataset. After removing the first and second degree relatives, 758 samples remained for further analyses (see Additional file 10). We used PLINK software 1.05 [93] to filter the combined dataset to include only SNPs on the 22 autosomal chromosomes with minor allele frequency >1% and genotyping success >97%.

### Analyses of genome-wide SNP data

We calculated mean pairwise F_ST_ values between populations for 513440 autosomal SNPs by using the method of Weir and Cockerham [94] assembled into a R script [95]. Only populations with N ≥ 4 were included in the F_ST_ calculation. Given the high level of European admixture in some Siberian and American populations, resulting from recent gene flow from Europe, the interpretation of F_ST_ distances between samples from different, although genetically closely related, populations might not necessarily be straightforward. To validate our results, we recalculated F_ST_ values excluding very mixed samples. The final data set consisted of 695 individuals (see Additional file 10). Because background linkage disequilibrium (LD) can affect both principal component and structure-like analysis [96], we thinned the dataset by removing one SNP of a pair in strong LD (genotypic correlation r^2^>0.4) in a window of 200 SNPs (sliding the window by 25 SNPs at a time). The final dataset consisted of 202895 SNPs that were used in subsequent analyses. PCA was carried out using the smartpca program of the EIGENSOFT package [96]. ADMIXTURE [50], which implements a structure-like [97] model-based maximum likelihood (ML) clustering algorithm, was used to assess population structure via inferring the individual ancestry proportions. To monitor convergence between individual runs, we ran ADMIXTURE one hundred times at K = 2 to K = 14. At low values of K, all runs arrive at the same or very similar Loglikelihood scores (LLs), whereas at high K-s the LLs vary. However, judging by the low level of variation in LLs (LLs < 1) within a fraction (10%) of runs with the highest LLs, we assume that the global loglikelihood maximum was reached at K = 2 to K = 7 and K = 11 (see Additional file 12A). ADMIXTURE provides an assessment of the “best” K by computing a cross validation index, which points to the predictive accuracy of the model at a given K. In our setting the best predictive power was observed at K = 6 (see Additional file 12B).

### Mantel test

The correlation of genetic, linguistic, and geographic distances was assessed by the Mantel test, employing the Arlequin 3.01 software package [98] with 100,000 permutations. To test whether statistically significant associations between linguistic and genetic affiliations reflect the same events in population history or parallel, but separate isolation by distance processes, we performed partial correlations, keeping geography constant [99]. The Weir and Cockerham [94] pairwise average F_ST_ matrix was used in case of autosomal SNP data. When the Mantel test was applied to mtDNA and Y chromosome data, genetic distances were based on Slatkin´s linearized F_ST_s. Geographic distances between populations were calculated from a list of geographic coordinates of the sampled sites by using the Geographic Distance Matrix Generator (version 1.2.3) [100]. The relationships between the languages of all pairs of populations were classified according to the following numerical scheme: 0, same languages; 1, languages in the same branch of a family; 2, languages in different branches of the same family; 3, languages in different families. The branches were defined as major subfamilies that diverge close to the root of the family tree. The arbiter for linguistic classification was the online version of the [51].

### Availability of supporting data

21 novel complete mtDNA sequences supporting the results of this article are available in the National Center for Biotechnology Information [Genbank], [accession numbers KC985147-KC985167, http://www.ncbi.nlm.nih.gov/Genbank/].

The genome-wide SNP data generated for this study are available in the National Center for Biotechnology Information – Gene Expression Omnibus [NCBI GEO], [dataset nr. GSE46828, http://www.ncbi.nlm.nih.gov/geo/query/acc.cgi?acc=GSE46828] and in PLINK format in our web at http://www.ebc.ee/free_data.

## Competing interests

The authors declare that they have no competing interests.

## Authors’ contributions

RV and SAF conceived and designed the study. MIT, EKK, FAP, SAF, MIV and LPO coordinated and conducted the field study. AT participated in the coordination of mtDNA study. SAF, MR and SR carried out the mtDNA and Y chromosome genotyping. SIZ performed genotyping of Dolgan mtDNA from southern Taymyr. MR, EM, NT, BHK and AO performed full sequencing of mtDNA. MR, SAF, KT and MM analyzed the data. MR and SAF wrote the manuscript. MM revised the manuscript focusing on autosomal data. All authors read and approved the final manuscript.

## Supplementary Material

Additional file 1 Genotyping information for 701 mtDNAs from five native populations of Sakha and 128 mtDNAs from Dolgans in Taymyr. Click here for file

Additional file 2 **Phylogenetic tree based on 80 complete mtDNA sequences from haplogroup Z.** Mutations relative to the RSRS [35] are indicated on the branches. Capital letters are used for transitions and lowercase letters for transversions. Heteroplasmies are labeled using the IUPAC code and capital letters (e.g., 73R). Recurrent mutations are underlined. Reversal mutations are suffixed with “!”. Insertions are indicated by a dot followed by the position number and type of inserted nucleotide(s). Deletions are indicated by a “d” after the deleted nucleotide position. The control-region sequence is not reported for the sample As30. For phylogeny construction, the highly variable site 16519 and the length variation in the poly-C stretches at nps 303-315 and 16184-16194 were not used. A-C transversions at nps 16182 and 16183 were excluded because of their dependence on the presence of the C-T transition at np 16189. The box containing the sample ID is color coded according to the geographic origin of the sample, and the accession number and/or the publication from which it was retrieved is denoted below the ID. Coalescence time estimates expressed in kilo years ago are shown next to clade labels and were calculated based on the rho statistic and standard deviation as in [59,90]. The calculator provided by [91] was used to convert the rho statistics and its error ranges to age estimates with 95% confidence intervals. Sample As30 was excluded from the calculations, as its control region is not reported. Click here for file

Additional file 3 Population frequencies of mtDNA Z sub-haplogroups. Click here for file

Additional file 4 **Phylogenetic tree based on 37 complete mtDNA sequences from haplogroup R1.** Mutations relative to the RSRS [35] are indicated on the branches. Capital letters are used for transitions and lowercase letters for transversions. Heteroplasmies are labeled using the IUPAC code and capital letters (e.g., 73R). Recurrent mutations are underlined. Reversal mutations are suffixed with “!”. Insertions are indicated by a dot followed by the position number and type of inserted nucleotide(s). Deletions are indicated by a “d” after the deleted nucleotide position. For phylogeny construction, the length variation in the poly-C stretches at nps 303-315 and 16184-16194 was not used. A-C transversions at nps 16182 and 16183 were excluded because of their dependence on the presence of the C-T transition at np 16189. The box containing the sample ID is color coded according to the geographic origin of the sample, and below it the accession number and/or the publication from which it was retrieved is denoted. Coalescence time estimates expressed in kilo years ago are shown next to clade labels and were calculated based on the rho statistic and standard deviation as in [59,90]. The calculator provided by [91] was used to convert the rho statistics and its error ranges to age estimates with 95% confidence intervals. Sample Azeri10 was excluded from the calculations because of multiple heteroplasmic sites in the sequence. Click here for file

Additional file 5 **Frequencies of Y-STR haplotypes in the native populations of Sakha.** Designations of populations are as in Figure 1. Sample sizes are given in parentheses. Click here for file

Additional file 6 Y-STR haplotypes of 398 samples from haplogroup N1c. Click here for file

Additional file 7 Y-STR haplotypes of haplogroup C3* in the native populations of Sakha. Click here for file

Additional file 8 Phylogenetic network of the Y-chromosome haplogroup C3*. This median joining network of C3* haplotypes was constructed by employing STR data (11 loci: DYS385a, DYS385b, DYS389I, DYS389II, DYS390, DYS391, DYS392, DYS393, DYS437, DYS438 and DYS439) from 121 individuals using the program Network 4.6.1.0. Circles represent microsatellite haplotypes, the areas of the circles and sectors are proportional to haplotype frequencies according to the data presented in Additional file 9. Populations from Sakha and the linguistic affiliations of the rest of the samples are indicated by color. Click here for file

Additional file 9 Y-STR haplotypes of 121 samples from haplogroup C3*. Click here for file

Additional file 10 Details of samples included in autosomal SNP data analyses. Click here for file

Additional file 11 **ADMIXTURE plots from K = 2 to K = 14.** At each K the run with the highest log-likelihood of 100 runs is plotted. Each vertical column corresponds to one sample and represents its probability to have ancestry in the constructed ancestral populations differentiated by colors. Click here for file

Additional file 12 **ADMIXTURE analysis from K = 2 to K = 14.** a) log-likelihood scores (LLs) of all the 14 × 100 runs of ADMIXTURE. Inset shows the extent of this variation in the fractions (5%, 10%, 20%) of runs that reached the highest LLs. b) Box and whiskers plot of the cross validation indexes of all 1400 runs of ADMIXTURE. Click here for file

Additional file 13 **ADMIXTURE plots at K=13.** Ten runs with the highest log-likelihood were plotted. Click here for file

Additional file 14 Correlation and partial correlation coefficients, r (P-values), between genetic, geographic, and linguistic distances. Click here for file

Additional file 15 Additional information on the mtDNA and Y chromosome data used in the Mantel test. Click here for file

Additional file 16 Additional information on the native populations of Sakha. Click here for file
