# Supplementary material for: Autosomal and uniparental portraits of the native populations of Sakha (Yakutia): implications for the peopling of Northeast Eurasia
Source: BMC Evol Biol. 2013 Jun 19;13:127. doi: 10.1186/1471-2148-13-127 (PMC3695835; doi:10.1186/1471-2148-13-127)
Supplement: Additional file 8 — Phylogenetic network of the Y-chromosome haplogroup C3*. This median joining network of C3* haplotypes was constructed by employing STR data (11 loci: DYS385a, DYS385b, DYS389I, DYS389II, DYS390, DYS391, DYS392, DYS393, DYS437, DYS438 and DYS439) from 121 individuals using the program Network 4.6.1.0. Circles represent microsatellite haplotypes, the areas of the circles and sectors are proportional to haplotype frequencies according to the data presented in Additional file 9. Populations from Sakha and the linguistic affiliations of the rest of the samples are indicated by color. [file 1471-2148-13-127-S8.pdf]

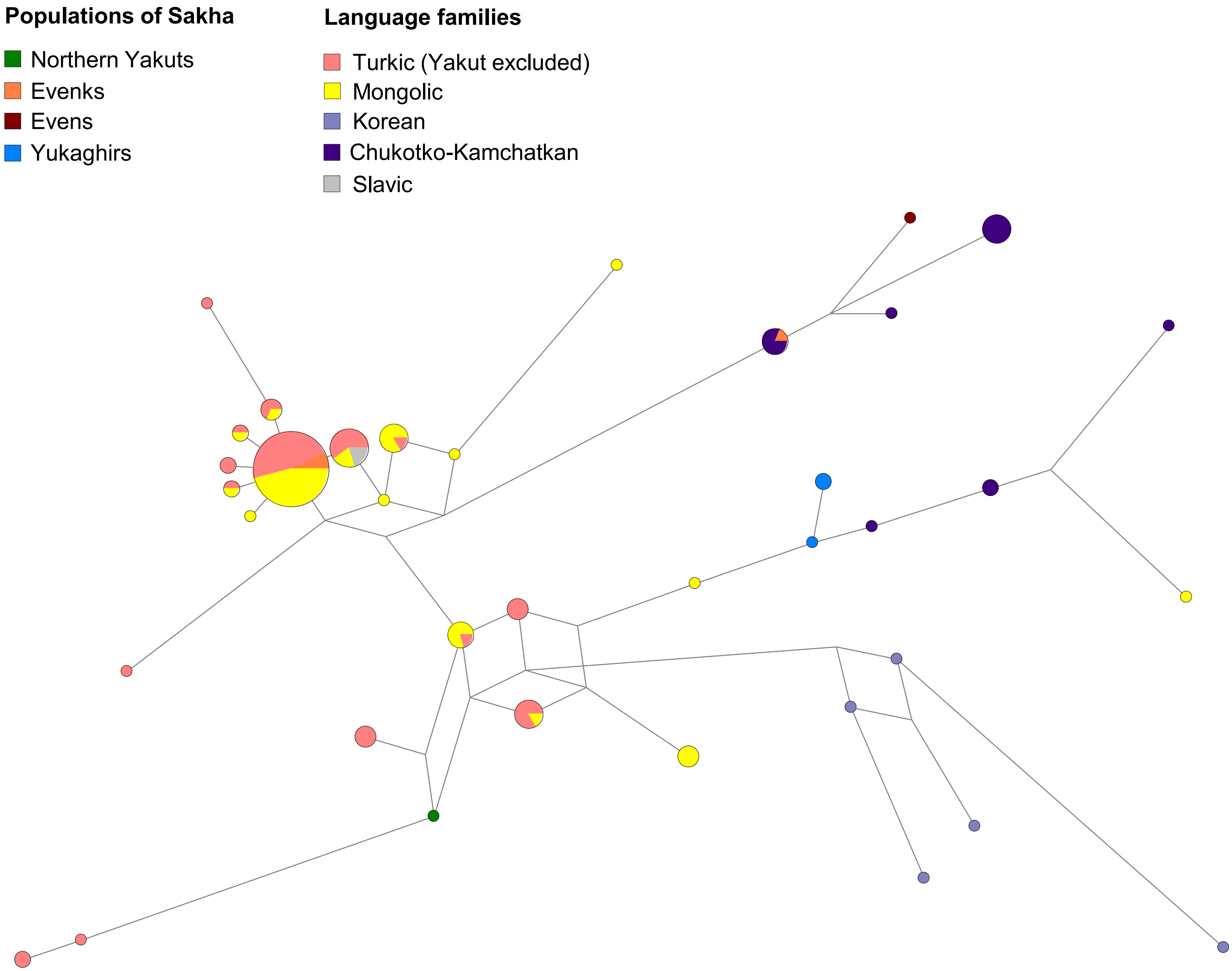
Populations of Sakha
Northern Yakuts
Evenks
Evens
Yukaghirs
Language families
Turkic (Yakut excluded)
Mongolic
Korean
Chukotko-Kamchatkan
Slavic
