# Supplementary figures and images for: Autosomal and uniparental portraits of the native populations of Sakha (Yakutia): implications for the peopling of Northeast Eurasia
Source: BMC Evol Biol. 2013 Jun 19;13:127. doi: 10.1186/1471-2148-13-127 (PMC3695835; doi:10.1186/1471-2148-13-127)

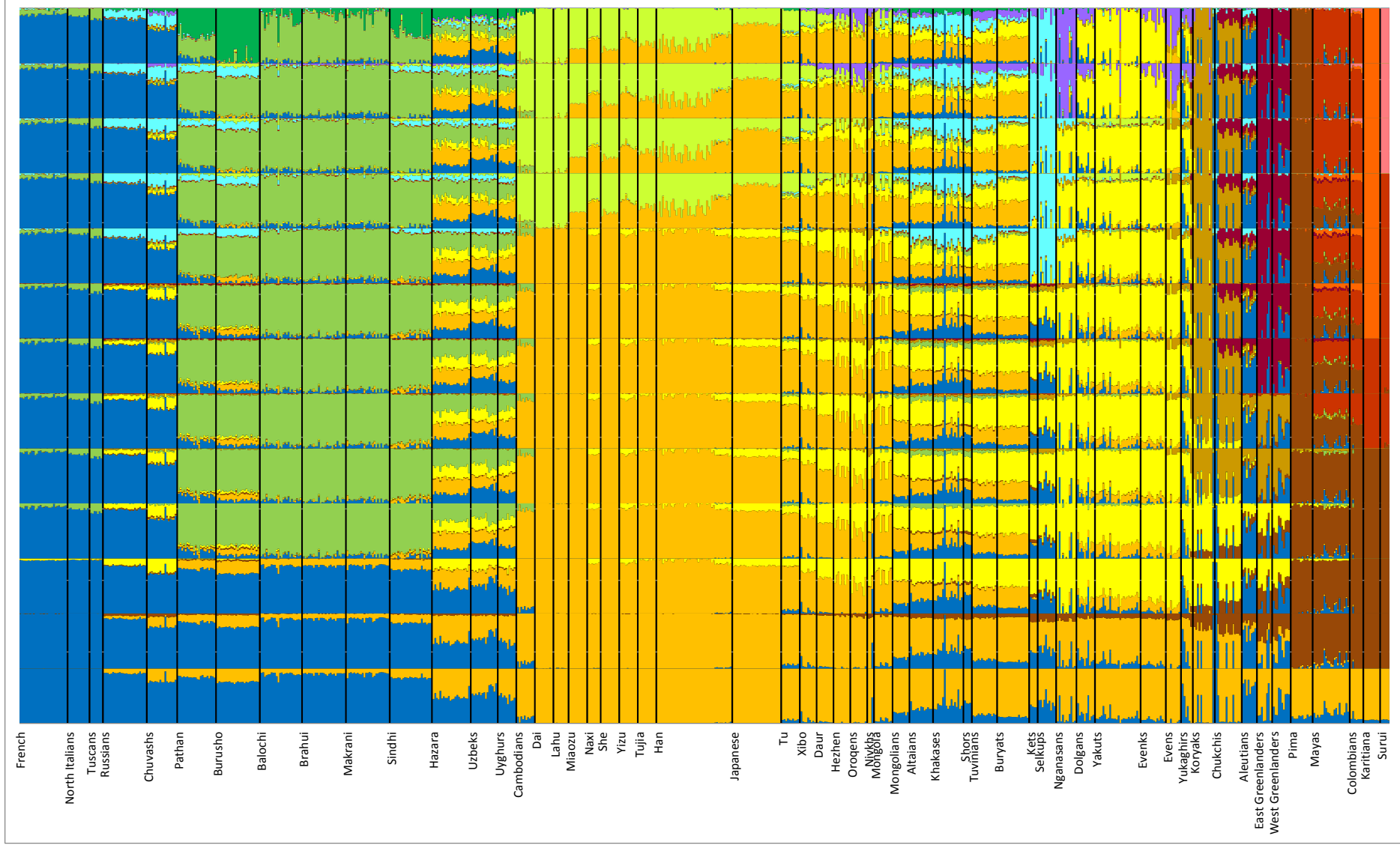

Supplement: Additional file 11 — ADMIXTURE plots from K = 2 to K = 14. At each K the run with the highest log-likelihood of 100 runs is plotted. Each vertical column corresponds to one sample and represents its probability to have ancestry in the constructed ancestral populations differentiated by colors. [file 1471-2148-13-127-S11.pdf]

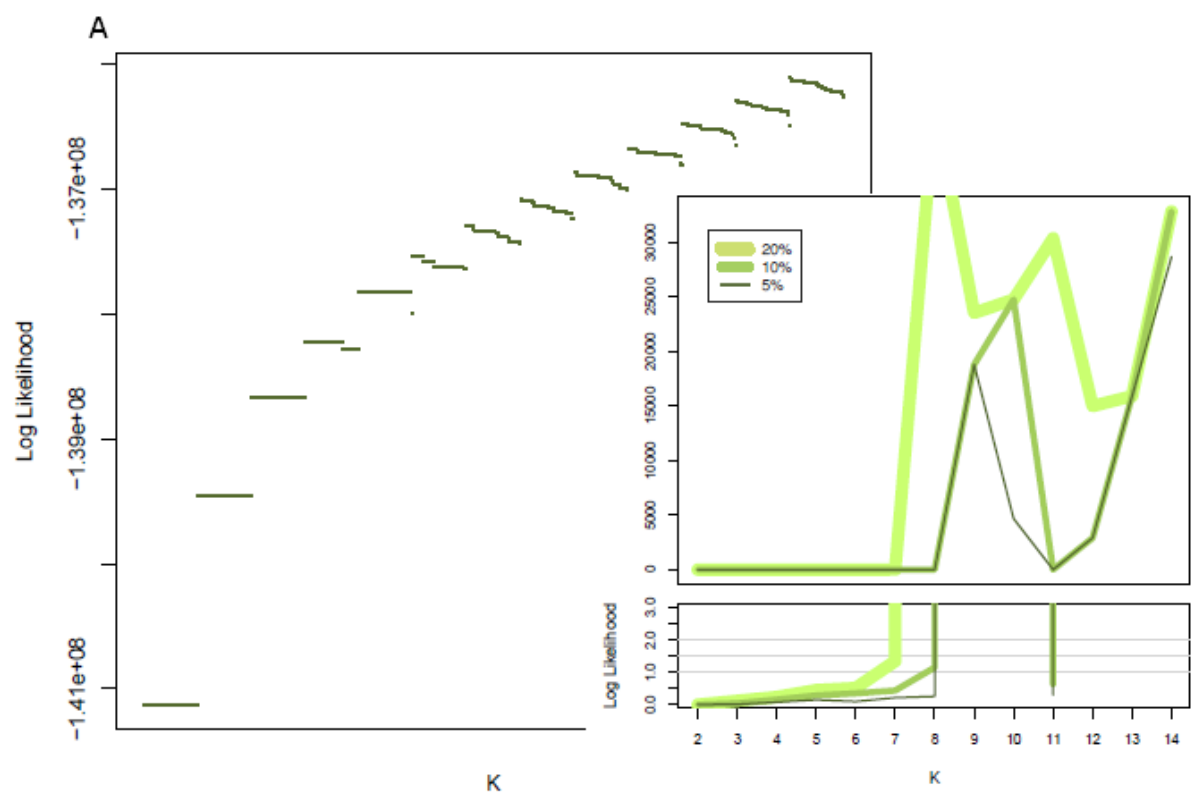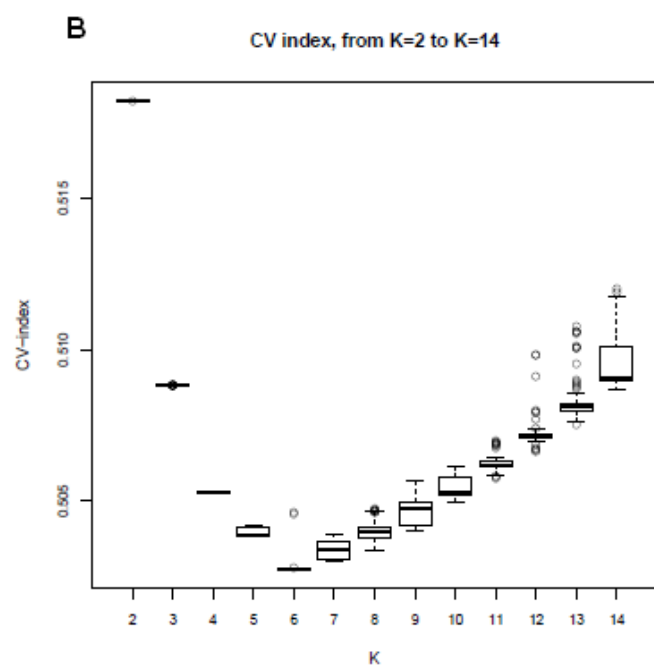

Supplement: Additional file 12 — ADMIXTURE analysis from K = 2 to K = 14. a) log-likelihood scores (LLs) of all the 14 × 100 runs of ADMIXTURE. Inset shows the extent of this variation in the fractions (5%, 10%, 20%) of runs that reached the highest LLs. b) Box and whiskers plot of the cross validation indexes of all 1400 runs of ADMIXTURE. [file 1471-2148-13-127-S12.pdf]

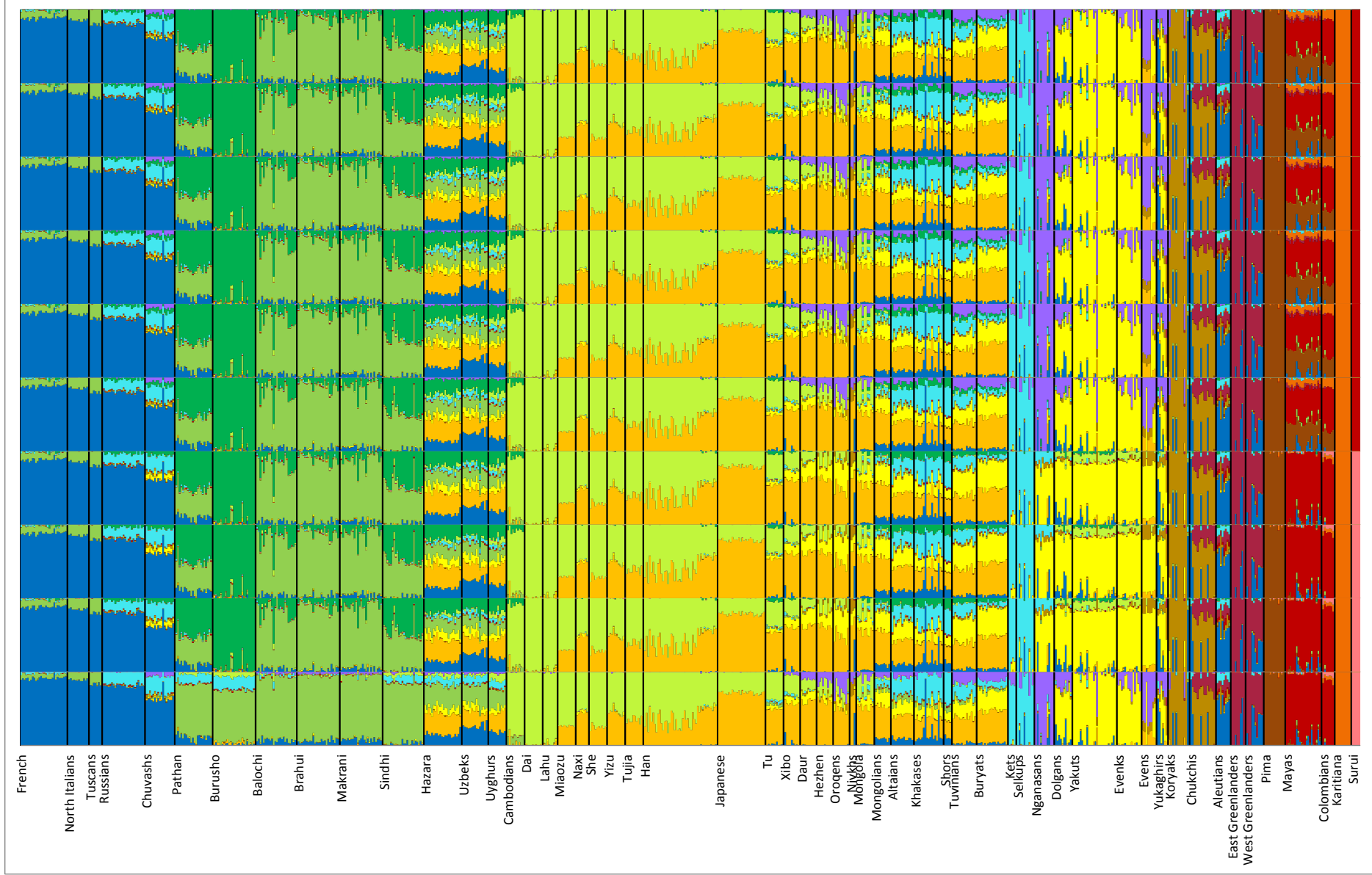

Supplement: Additional file 13 — ADMIXTURE plots at K=13. Ten runs with the highest log-likelihood were plotted. [file 1471-2148-13-127-S13.pdf]
