# Supplementary material for: Autosomal and uniparental portraits of the native populations of Sakha (Yakutia): implications for the peopling of Northeast Eurasia
Source: BMC Evol Biol. 2013 Jun 19;13:127. doi: 10.1186/1471-2148-13-127 (PMC3695835; doi:10.1186/1471-2148-13-127)
Supplement: Additional file 16 — Additional information on the native populations of Sakha. [file 1471-2148-13-127-S16.pdf]

**Additional file 16. Additional information on the native populations of Sakha.**

| Population | Linguistic affiliation | Census size at 2010[1] | | Sample size | |
|---|---|---|---|---|---|
| | | in Russia | in Sakha | mtDNA | NRY |
| Central Yakuts | Altaic (Turkic) | 478 085[2] | 466 492[2] | 164 | 92 |
| VilyuyYakuts | Altaic (Turkic) | | | 111 | 58 |
| Northern Yakuts | Altaic (Turkic) | | | 148 | 66 |
| Evenks | Altaic (Tungusic) | 38 396 | 21 008 | 125 | 57 |
| Evens | Altaic (Tungusic) | 21 830 | 15 071 | 105 | 24 |
| Yukaghirs | *Isolate (Yukaghir) | 1 603 | 1 281 | 22 | 11 |
| Dolgans | Altaic (Turkic) | 7 885 | 1 906 | 26 | 10 |

[1]data from [1].

[2]census size of all Yakuts.

*Yukaghir is classified as a language isolate by [2], but as a member of Uralic-Yukaghir language family by [3].

## References


1. **Окончательные итоги Всероссийской переписи населения 2010 года** [http://www.perepis-2010.ru/results_of_the_census/results-inform.php]

2. Lewis MP: *Ethnologue: Languages of the World*. 16th edition. Dallas: SIL International; 2009.

3. Ruhlen M: *A Guide to the World's Languages: Classification*. Stanford: Stanford University Press; 1987.
